# Data-driven analysis of the number of Lennard–Jones types needed in a force field

**DOI:** 10.1038/s42004-020-00395-w

**Published:** 2020-11-13

**Authors:** Michael Schauperl, Sophie M Kantonen, Lee-Ping Wang, Michael K Gilson

**Affiliations:** 1 Skaggs School of Pharmacy and Pharmaceutical Sciences, 9500 Gilman Drive, MC0751, University of California, San Diego, CA 92093-0751 USA; 2 Department of Chemistry, University of California, Davis, CA 95616 USA

**Keywords:** Method development, Computational chemistry

## Abstract

Force fields used in molecular simulations contain numerical parameters, such as Lennard–Jones (LJ) parameters, which are assigned to the atoms in a molecule based on a classification of their chemical environments. The number of classes, or types, should be no more than needed to maximize agreement with experiment, as parsimony avoids overfitting and simplifies parameter optimization. However, types have historically been crafted based largely on chemical intuition, so current force fields may contain more types than needed. In this study, we seek the minimum number of LJ parameter types needed to represent the key properties of organic liquids. We find that highly competitive force field accuracy is obtained with minimalist sets of LJ types; e.g., two H types and one type apiece for C, O, and N atoms. We also find that the fitness surface has multiple minima, which can lead to local trapping of the optimizer.

## Abstract (web summary)

The force fields used in molecular simulations typically assign varied Lennard-Jones parameters to the atoms of a given element, depending on the neighborhood of each atom in the chemical graph of a compound, but optimizing so many parameters without overfitting is challenging. Here, the authors provide evidence that far fewer LJ parameters may be needed.

## Introduction

Molecular simulations are a widely used tool to study biological and chemical questions at an atomistic level^1,2^. Applications include modeling of protein–protein interactions^3^; protein folding^4^; the interactions of nucleic acids^5–7^; and the binding of small molecules by proteins, with applications to drug design^8,9^. Large systems, such as biomolecules in solution, pose substantial computational challenges. Improved hardware and sampling algorithms have significantly improved the ability of simulations to sample phase space^10–12^. Nonetheless, simulation results still can deviate significantly from experimental measurements^13,14^, and the underlying potential functions, also known as force fields (FF), are thought to be an important source of error^15^. Indeed, the accuracy of molecular simulations is necessarily limited by the accuracy of the FF.

There are two broad approaches to improving the accuracy of FFs. One approach is based on the recognition that most biomolecular FFs have for decades used the same functional form, which comprises harmonic bond-stretches and angle-bends, sinusoidal torsional terms, Lennard–Jones (LJ) interactions to model the van der Waals forces, and atom-centered point charges to model the electrostatics. Changing this functional form to one which can capture the physics in greater detail is a promising route to improve FF accuracy. Improved functional forms may include new terms, e.g., to capture polarization more accurately^16–18^, or replace old terms with newer more accurate ones, e.g., 6–12 LJ potential with a new exp-6 form^19^. The second approach to improving FFs is to keep the simplistic form of common biomolecular FFs and instead improve the selection of the FF’s adjustable parameters, such as torsional barriers, partial charges, and LJ radii and well-depths. This can be done by including more, and more relevant, training data in the parameterization process. For example, the inclusion of host guest binding data is a promising route to train a force field to accurately describe molecular interactions^20–25^. In addition, new parameterization protocols for old force field terms, e.g., point charges, may be developed^26,27^.

When improving the parameterization of a FF, it is possible to adjust not only the numerical parameters assigned to each FF type — such as the LJ parameters assigned to carbonyl oxygens – but also the type definitions themselves. For example, every carbonyl oxygen may be assigned the same LJ parameter, or, alternatively, distinct LJ parameters may be assigned to subsets of a functional group, such as the carbonyl oxygen in an amide group. Such type definitions have historically been based largely on chemical intuition and by analysis of specific simulation results^28^. This has led to an increasing number of FF types as new parameters were added in an arguably ad hoc manner to solve perceived problems. A recent effort to streamline FF typologies by replacing atom-typing with direct chemical perception has led to a FF with markedly fewer independent parameters^28^, but there are still 35 different LJ types and hence 70 LJ parameters.

The increased computational power available in recent years allows us now to adjust parameters in a more systematic way, and new tools have been developed to automate this process^29,30^. However, parameter optimization is still challenging, as the large number of FF types and hence of independent parameters means these calculations are generally subject to the curse of dimensionality. This holds especially for LJ parameters, which are usually trained and tested against condensed phase data, so that time-consuming molecular dynamics (MD) simulations must be run within the optimization loop. In addition, a data-driven approach that would allow automated deletion, addition, and modification of FF types as part of the optimization process has not yet been reported, though there have been initial steps in this direction^31^. As a consequence, the type definitions of LJ types have not changed by much.^32–36^.

Two approaches to reducing the complexity of LJ parameter assignments may be considered. One derives bespoke LJ parameters for a molecule of interest via an atoms-in-molecules analysis of its electronic structure^37,38^. This approach avoids high-dimensional optimization by using only a few adjustable parameters that control the mapping from electronic structure to the LJ parameters. The second approach is to sharply reduce the number of LJ types. In the context of our effort to generate an improved version of the restrained electrostatic potential (RESP)^39^ partial charge method, we optimized a FF that allowed only five LJ types, one each for carbon, nitrogen, and oxygen, and two for hydrogen^27^. The accuracy reached by this simplified model surprised us and led us to ask whether FFs really need all the LJ types which are usually used, or whether, instead, similar accuracy can be achieved with far fewer LJ types.

Accordingly, the present study explores the accuracy that can be achieved by FFs with highly reduced numbers of LJ types. We use ForceBalance to optimize the LJ parameters for chemical motivated LJ typologies against experimentally measured properties of pure organic liquids, and then test against a second set of experimental data. Results are generated for both RESP2^27^ and regular RESP partial charges^39^, and the robustness of the conclusions are further evaluated with additional runs using a larger training set and a different test set, and by comparisons with the baseline SMIRNOFF99Frosst-1.0.7^28^ (SmirFF.7) and generalized amber force field (GAFF) version1.8 force fields^32^. We find that minimalist LJ typing schemes, such as one with only two hydrogen types and one type each for carbon, oxygen, and nitrogen, perform as well as much more complex typing schemes. These results have intriguing implications for future FF development.

## Results

**Fig. 1:**
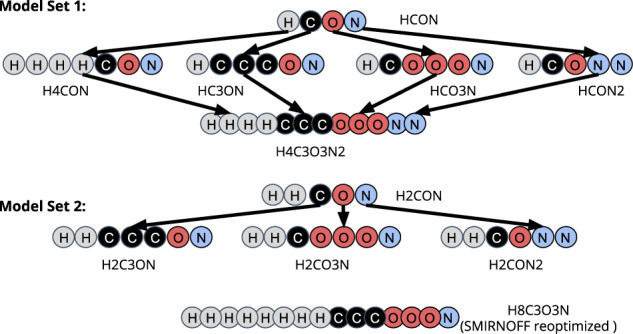
Summary of LJ typing models investigated in this study. Each group of elements represents one LJ model for which ε and r_1/2_ parameters were trained and evaluated. Arrows indicate a “more general → more specific” relationship between two typing models. Model Set 1 starts from the simplest model with only one LJ type per element and increases complexity one element at a time. Model Set 2 is similar but always distinguishes between polar and apolar hydrogens. The final model is a reoptimized version of SmirFF.7.

In the first subsection of the Results, we summarize the LJ models considered in this study. In the next subsection, we report on the training- and test-set performance of all of the LJ typing models depicted in Fig. 1, using RESP1 charges and both training set/test set splits (see Methods section). The third subsection then examines the complexity of the multidimensional LJ parameter optimization process. The fourth subsection explores the sensitivity of the results to the partial charges used by re-running the Training Set 1/Test Set 1 analysis with RESP2 charges^27^. The figures present results for the optimizations that gave the lowest values of the training-set objective function. The means and ranges over the triplicates are provided in the Supporting Information (Supplementary Data 1), as are the optimized LJ parameter sets (Supplementary Data 2). Because the objective function scales roughly with the number of data used, it runs higher for the test sets than for the smaller training sets. To put training- and test-set objective functions on similar scales in the figures, the Test Set 1 objective functions were scaled by a factor of 1/15 and the Test Set 2 objective functions were scaled by 1/3.

### LJ Models investigated in this study

Ideally, it would be possible to optimize not only the numerical LJ parameters associated with a predefined set of LJ types, but also the type classification itself. However, we are not aware of any software with this capability. Therefore, we instead propose various sets of chemically motivated LJ types and optimize the LJ parameters for each of these models. In total, we consider 11 different models, summarized in Fig. 1, where the models are abbreviated according to the number of LJ types for each element. For example, model H4CON has four H types and one each for C, O, and N, while the first model, HCON, has just one type per element. Starting from this minimal set, we first try allowing multiple types for a single element (Fig. 1, row 2). For example, we split the hydrogen LJ type into four (H bound to sp3 C, H bound to sp2 or sp C, H bound to N, and H bound to O) resulting in model H4CON, which has seven types. We also trained one model with all of the potential element types (Fig. 1, row 3), to generate model H4C3O3N3. Then, because it may be necessary to distinguish at least polar and apolar hydrogens in order to account for hydrogen-bonding, we repeated the procedure from above but always using only a polar and an apolar hydrogen LJ type. This results in the models H2CON, H2C3ON, H2CO2N, H2CON2 (Fig. 1, rows 4, 5). Finally, we also reoptimize LJ parameters for all the types in the current SmirFF.7^28^ that are represented in the training and test sets, amounting to 8 hydrogen types, 3, carbon types, 3 oxygen types, and 1 nitrogen type (Fig. 1, row 6).

### Performance of LJ typing models with RESP charges

**Fig. 2:**
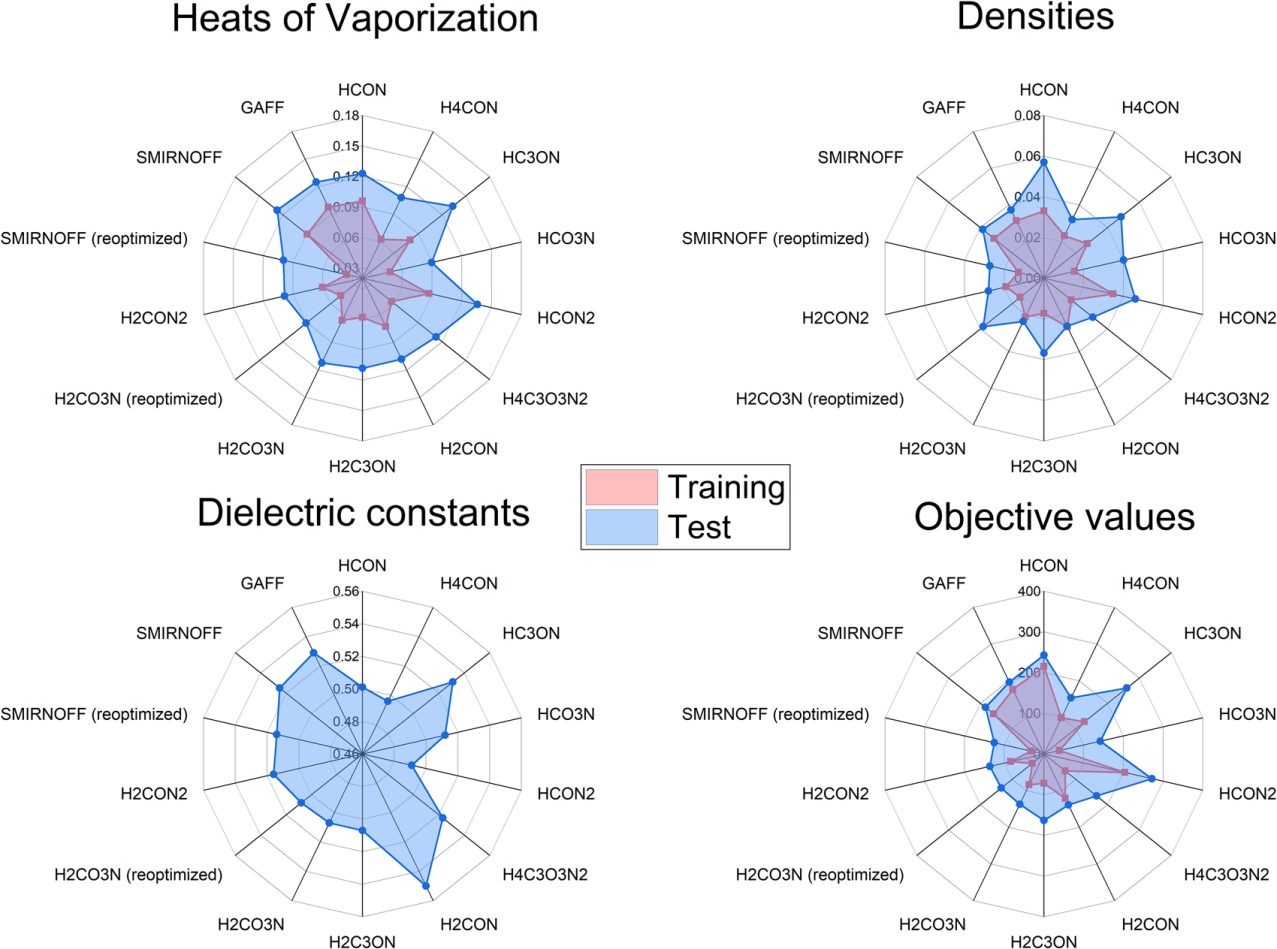
Relative errors for Training and Test Set 1. Relative errors for experimental data (heats of vaporization, densities, dielectric constants), and for the ForceBalance objective function, for Training Set 1 (red) and Test Set 1 (blue). The test set objective functions are scaled by 1/15 (see main text). The number of parameters may be deduced from names of the LJ models; e.g., HC3ON has 1 + 3 + 1 + 1 = 6 parameters.

**Fig. 3:**
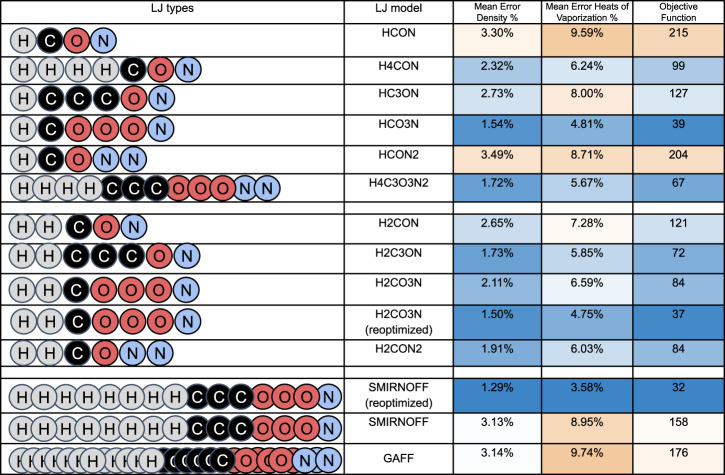
Training set results for Training Set 1 optimization of LJ parameters with RESP partial charges. Errors and objective function values are reported for the Training Set 1 compounds. These results are for the replicates that gave minimum training set values of the objective function for each model.

**Fig. 4:**
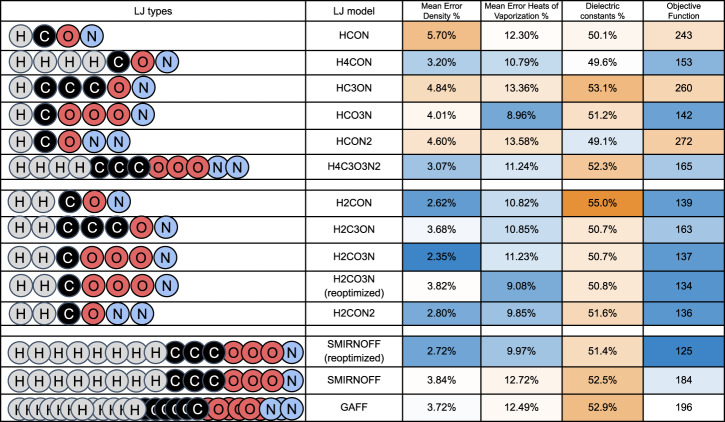
Test set results for Training Set 1 optimization of LJ parameters with RESP partial charges. Errors and objective function values are reported for the Test Set 1 compounds. Objective function values are scaled by 1/15 (see main text). These results are for the replicates that gave minimum training set values of the objective function for each model.

**Fig. 5:**
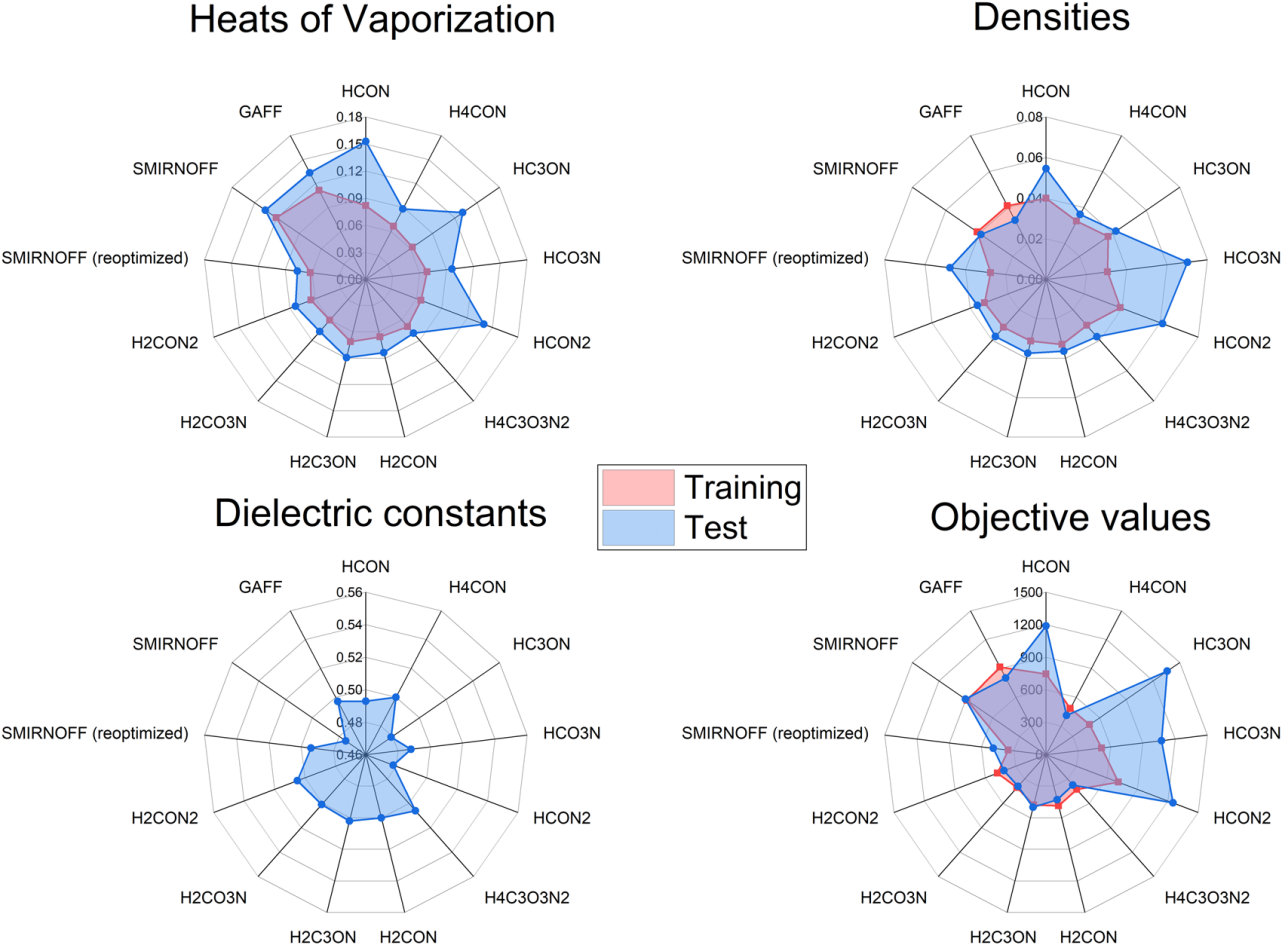
Relative errors for Training and Test Set 2. Relative errors for experimental data (heats of vaporization, densities, dielectric constants), and ForceBalance objective function, for Training Set 2 (red) and Test Set 2 (blue). The test set objective functions are scaled by 1/3 (see main text). The numbers of parameters may be deduced from name of the model; e.g., HC3ON has 1 + 3 + 1 + 1 = 6 parameters.

**Fig. 6:**
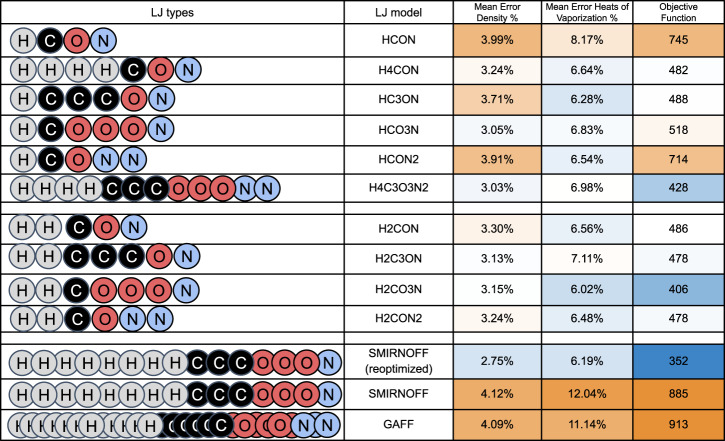
Training set results for Training Set 2 optimization of LJ parameters with RESP partial charges. Errors and objective function values are reported for the Training Set 2 compounds. These results are for the replicates that gave minimum training set values of the objective function for each model.

**Fig. 7:**
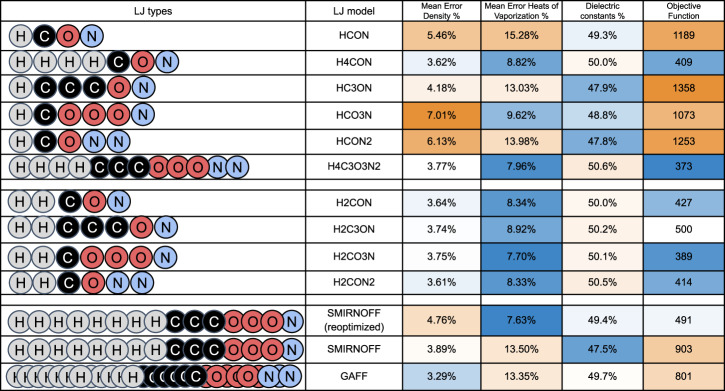
Test set results for Training Set 2 optimization of LJ parameters with RESP partial charges. Errors and objective function values are reported for the Test Set 2 compounds. Objective function values are scaled by 1/3 (see main text). These results are for the replicates that gave minimum training set values of the objective function for each model.

*A single LJ type per element affords competitive accuracy*: Perhaps unexpectedly, the simplistic HCON typing model yields test-set accuracy not much below that obtained with the original SmirFF.7LJ parameters, although it has only four LJ types, whereas SmirFF.7 has 15 for the training and test set compounds. Thus, following optimization with Training Set 1, the HCON model’s Test Set 1 errors for densities, heats of vaporization (HOVs), and dielectric constants are 5.70%, 12.30%, and 50.1%, respectively, while those of SmirFF.7 are 3.84%, 12.72%, and 52.5%; see Figs. 2–4. For the second training/test-set split (see Methods section), the accuracy of the HCON model (5.46%, 15.28%, 49.3%) is again slightly lower than that of SmirFF.7 (3.89%, 13.50%, 47.5%) (Figs. 5–7). The results for GAFF-1.8, with 28 atom types for these compounds, are similar to those for SmirFF.7, as shown in the bottom rows of these figures.

*Allowing polar and apolar H types improves accuracy*: The present study agrees with the expectation that distinguishing LJ types between polar and apolar hydrogens allows substantially greater accuracy^40^. Thus, going from HCON to H2CON allows the objective function to drop substantially for both training sets and both test sets and in fact to outperform both SmirFF.7 and GAFF-1.8 for all training and test-sets (Figs. 2–7). Similarly, splitting the single H type in HC3ON, HCO3N, and HCON2 into polar and apolar types to yield models H2C3ON, H2CO3N, and H2CON2, respectively, yields consistently lower values of the objective function for both test sets (Figs. 4 and 7). However, none of these models yields convincingly better results than the simpler H2CON model.

Further splitting of hydrogen into four LJ types does not appear to be useful, as the results for H2CON and H4CON differ minimally for the test sets (Figs. 4 and 7). Moreover, given a split between polar and apolar hydrogen types, adding more carbon, oxygen, and nitrogen types does not significantly improve accuracy for either test-set (Figs. 4 and 7).

*Splitting H, O types is advantageous, but not C, N*: As expected, adding LJ types for any element to HCON to form models H4CON, HC3ON, HCO3N, and HCON2 leads to improved accuracy for both training sets, based on the overall objective function (Figs. 3 and 6). However, although adding H and O types additionally leads to improved test-set accuracy, adding C and N types leads to unchanged or worse test-set accuracy (Figs. 4 and 7). Thus, the gains in training set accuracy on adding carbon and nitrogen types may be due to overfitting. Interestingly, increasing the number of oxygen types from one to three, while keeping the number of hydrogen types fixed, consistently leads to improved accuracy for heats of vaporization (HOV), though not for density or dielectric constant.

*Optimizations can terminate at multiple local minima*: Any LJ typing model that can be derived from a simpler model by splitting one or more of its LJ types should, upon optimization, be able to reach at least as low a value of the objective function for the training set. In practice, however, this is not consistently observed. For example, the Training Set 1 objective function of HCO3N is lower than that of two models with more adjustable parameters, H2CO3N and H4C3O3N2 (Fig. 3). We conclude that at least some of these optimizations are terminating at local, rather than global, minima. (It is also interesting that the optimized H4C3O3N2 model yields somewhat worse results than simpler models, for both test sets (Figs. 4, 7).) Further details regarding the challenges of optimizing larger numbers of LJ parameters are provided in the subsection dealing with sensitivity to initial parameter values.

*Reparameterization of SMIRNOFF types gives good accuracy*: We used the present training sets to reoptimize parameters for the full set of 15 LJ types associated with these compounds and SMIRNOFF force field. This led to the lowest objective functions obtained for either training set (Figs. 3 and 6). However, for the test sets, other models with far fewer LJ types, such as H2CON, yield similar or better test-set results (Figs. 4 and 7). Thus, the present data do not indicate a need for 15 LJ types.

### LJ parameter optimization is sensitive to initial values

As noted above, we observed several instances where the optimization of one LJ model led to lower (better) values of the training-set objective function than optimization of another LJ model where one or more types from the first had been split into multiple independent parameters. This means that the model with more parameters was not fully optimized, as the parameter space of the more complex is a superspace of the parameter space of the simpler model. Closer examination of one such case offers insight into how the optimization can be trapped at a local optimum. We focus on the optimization of model H2CO3N with Training Set 1 and RESP charges, which led to a higher (worse) value of the objective function (84) than that reached by the corresponding optimization of HCO3N (39).

We start by comparing optimized parameters for HCO3N and H2CON. The hydrogen parameters for HCO3N (Table 1) are similar to those of an aliphatic hydrogen, but its three sets of LJ oxygen parameters are quite distinct from each other (Table 1). In comparison, H2CON assigns much smaller radii to polar hydrogens than to apolar hydrogens (Table 1), but its oxygen parameters remain similar to those of the initial carbonyl oxygen. The initial optimization of H2CO3N (H2CO3N in Fig. 3) yields a clear distinction between small polar hydrogens and large apolar hydrogens, while assigning all three oxygen types rather similar parameters (Table 1). In effect, this optimization yielded parameters for H2CO3N that are more similar to those of H2CON than to those of HCO3N and that yield a value of the objective function (84) between those of H2CON (121) and HCO3N (39) (Fig. 3).

**Table 1:** Optimized LJ parameters for various LJ typing models. ε: kcal mol^−1^, r_1/2_: Å. See the text for details.

	HCON		HCO3N		H2CON		H2CO3N		H2CO3Nreopt	
SMIRKS	ε	r_1/2_	ε	r_1/2_	ε	r_1/2_	ε	r_1/2_	ε	r_1/2_
Hydrogen										
[#1:1]-[#6X4]	0.017	1.27	0.034	1.42	0.014	1.39	0.023	1.42	0.030	1.42
[#1:1]-[#6X3]	0.017	1.27	0.034	1.42	0.014	1.39	0.023	1.42	0.030	1.42
[#1:1]-[#7]	0.017	1.27	0.034	1.42	0.015	0.69	0.007	0.88	0.031	1.44
[#1:1]-[#8]	0.017	1.27	0.034	1.42	0.015	0.69	0.007	0.88	0.031	1.44
Carbon										
[#6:1]	0.074	2.11	0.077	1.84	0.081	2.06	0.078	1.96	0.078	1.87
[#6X4:1]	0.074	2.11	0.077	1.84	0.081	2.06	0.078	1.96	0.078	1.87
[#6X2:1]	0.074	2.11	0.077	1.84	0.081	2.06	0.078	1.96	0.078	1.87
Nitrogen										
[#7X3:1]	0.194	1.47	0.231	1.79	0.177	1.80	0.210	1.83	0.245	1.78
[#7:1]	0.194	1.47	0.231	1.79	0.177	1.80	0.210	1.83	0.245	1.78
Oxygen										
[#8:1]	0.220	1.11	0.137	1.73	0.205	1.61	0.197	1.71	0.100	1.65
[#8X2H1 + 0:1]	0.220	1.11	0.494	1.89	0.205	1.61	0.218	1.60	0.533	1.87
[#8X2H0 + 0:1]	0.220	1.11	0.087	1.82	0.205	1.61	0.151	1.59	0.096	1.82

We conjectured that starting the H2CO3N optimizing with distinct polar/apolar hydrogen parameters led to trapping of the parameter search in a local optimum with a prominent split between polar and apolar hydrogen parameters and a reduced split among the oxygen types. To test this conjecture, we reoptimized the parameters for H2CO3N, this time setting the initial parameters for all-atom types to the optimized parameters for HCO3N, with its prominent split of oxygen-type parameters (Table 1). Thus, the initial parameters for both polar and apolar hydrogen were the optimized parameters of H in HCO3N. The outcome was a set of parameters (H2CO3N reoptimized) with a training-set objective function of 37 (Fig. 3), widely split oxygen parameters as in the initial guess, and parameters for apolar and polar hydrogens that are similar to each other and to their starting guess (Table 1). Thus, we see significant dependence of the optimization results upon the starting guess, even for an LJ model with only seven types.

The concept that the initial optimization of H2CO3N is trapped in a local optimum may be elaborated by considering the Euclidean distances among the models considered in this section. To do this, we consider each model as a point in 24-dimensional parameter space, based on the 12 *r*_1/2_ values and 12 *ε* values for the SMIRKS strings in Table 1. To put the values of *r*_1/2_ and *ε* on an equal footing, we scaled up the values of ε by $\frac{{\overline {r_{1/2}} }}{{\overline {\it{ \in }} }} = 13.7$, the ratio of the mean of *r*_1/2_ to that of *ε*. (See Supplementary Table 1). The distances among the models in Table 1 are given in the form of a matrix in Table 2. One may see that H2CO3N is closest to H2CON, with its split hydrogen types, while H2CO3Nreopt is closest to HCO3N, with its split oxygen types, as anticipated.

**Table 2:** Matrix of Euclidean distances between the models listed in Table 1. Only the upper triangle is shown, because the matrix is symmetric, and the diagonal elements are identically zero.

	HCON	HCO3N	H2CON	H2CO3N	H2CO3Nreopt
HCON		4.63	1.39	1.63	5.19
HCO3N			4.67	4.12	0.81
H2CON				1.08	5.27
H2CO3N					4.73
H2CO3Nr					

As noted in the Methods Section, the ForceBalance objective function includes a weak restraint, which prevents parameters from straying very far from the initial parameter values. We considered whether this restraint might have prevented the first H2CO3N optimization from reaching the optimal parameters with split oxygen parameters and similar hydrogen parameters (i.e., H2CO3Nreopt). To do this, we used ForceBalance to compute the contribution of the restraint from the first optimization that would have been associated with the H2CO3Nreopt parameters. This value, 1.8, would not have been enough to make the objective function at the H2CO3Nreopt parameters higher than those at the H2CO3N parameters. Thus, the weak restraint does not appear to account for the optimizer’s having missed the lower minimum at the H2CO3Nreopt parameter set.

Nonetheless, to further test whether the restraints might have been responsible for introducing a barrier between the apparent local optima, we reran the initial H2CO3N calculation without any parameter restraints at all (*w*_*reg*_ = 0.0). This optimization again led to a local optimum with a training objective function > 80, similar to the initial H2CO3N calculation with restraints (*w*_*reg*_ = 0.1, training objective function 84). We conclude that at least two valid local optima exist and that which one is discovered depends on the parameters used to start the optimization run.

### LJ typing models in the context of RESP2 charges

We tested the robustness of these findings to the choice of partial charges by retraining all LJ models against Training Set 1, this time with partial charges generated with the RESP2 method. The RESP2 partial charge model is similar to RESP1, but it uses higher-level electronic structure calculations and provides a mixing parameter that allows empirical adjustment of the overall polarity of the model by scaling between gas-phase and aqueous-phase charges^27^. Here, we set the mixing parameter to 0.5, corresponding to equal contributions from these two phases. As detailed in Supplementary Figs. 1–3, many of the same patterns are observed:

- Adding more H and O types allow significantly improved test-set accuracy but adding more C and N-type does not.
- Splitting H into apolar and polar types consistently improves test-set accuracy.
- The parameters for model H4C3O3N2 are not fully optimized, as simpler models reach lower (better) values of the objective function for the training set.
- Reoptimization of the full set of SMIRNOFF parameters generates a model that is similar in accuracy to at least one simpler model.

The overall accuracy obtained with these RESP2 charges is similar to that obtained with RESP charges. As seen before^27^, RESP2 charges tend to give somewhat more accurate dielectric constants (improvements up to about 20%) and RESP1 charges give somewhat more accurate densities and HOVs. The results for the objective function are generally higher for RESP2 than for models with RESP1, as we decided to weigh the contribution of the dielectric constant very little, since its calculated values have larger numerical uncertainties. As previously discussed, the mixing parameter affords a simple way of tuning RESP2 charges and should, ideally, be co-optimized with the LJ parameters when deriving a force field.

## Discussion

The central finding of this study is that highly competitive force field accuracy can be obtained with minimalist sets of LJ types. For example, merely splitting apolar from polar hydrogens, while using one type apiece for carbon, oxygen, and nitrogen, yields a model that meets or exceeds the accuracy of SmirFF.7 and GAFF-1.8 for the present test sets. This data-driven observation arguably challenges a chemical intuition that distinct LJ types are needed for atoms in distinct functional groups. We also found that the proliferation of LJ types does not necessarily increase accuracy and can even degrade it, presumably due in part to overfitting. Taken together, these results suggest that de novo efforts at FF parameter optimization should start with a minimal set of LJ types, such as the H2CON, HCO3N, or H2CO3N models. Added type distinctions may then be tested, but they should be discarded if not supported by the data. This approach should avoid overfitting and speed the optimization process by minimizing the dimensionality of the search space. As paraphrased from Einstein, “Everything should be made as simple as possible, but no simpler.”^41^.

If one starts with a simple LJ typing model, such as H2CON, a natural way to increment its complexity in a data-driven manner is to use a greedy optimization approach: find a type split (e.g., O to O3) that improves results, lock it in, then find the next split that improves results and lock it in; etc. This approach would have worked well in the present setting, where one might progress from splitting polar and apolar hydrogens to then splitting oxygens, thus reaching perhaps the best model found here, H2CO3N. However, it is possible that two type splits under consideration could increase accuracy when used together, but not when used individually. In such cases, the greedy approach can fail to find the best typing model, because it considers each split only in isolation and therefore will never accept either one and so, in turn, cannot discover the benefit of splitting both types. It is not known how commonly this situation will occur in the present setting.

Another challenging question, which we have not addressed in this work, is how to decide what types should be tried in the first place. For example, although we split oxygens into hydroxyls, ethers, and carbonyls, we could instead have lumped together with the ethers and carbonyls and thus considered only two types. Or, we might have tried an entirely different approach, setting up LJ types based on some measure of polarity, such as the partial charge assigned to each atom. Perhaps the ideal method would be to allow the types themselves to evolve in a purely data-driven manner, as previously explored with Monte Carlo sampling of FF typing schemes^28^, but that approach may still be too time-consuming for current use.

Also, the parameter optimization for a given LJ typing model has its own challenges. We observed that the steepest descent optimization method used here led to trapping of the optimizer in a local, rather than the global, optimum. This problem is expected to grow more challenging as the number of parameters increases, and there is always the possibility that a narrow optimum in a high dimensional parameter space will be missed. The chances of finding the global optimum may be increased by using multiple steepest descent optimizations at different initial points in the parameter space and/or by moving to one of the many global optimization strategies that have been developed over the years, such as genetic algorithms^42^ or Monte Carlo simulated annealing^43^. It should be noted, however, that such methods are likely to require considerably more steps than ForceBalance, and thus risk being overly time-consuming. The present analysis also points to a straightforward reality check for the adequacy of a parameter optimization: if a model with more finely divided types yields worse accuracy on the training set than another model where some of the finer types are lumped into coarser type divisions, then the more detailed model is not fully optimized. That said, if the finer model does yield better training set accuracy, this still cannot prove it is fully optimized. These warnings presumably also apply when LJ parameters are optimized along with other parameter types, such as bonded terms.

Starting from the one type per element model, HCON, it is of interest to consider which increases in complexity yield the greatest improvements inaccuracy. Perhaps the single most useful step is to distinguish between polar and apolar hydrogens, making model H2CON. This dramatically and consistently improves test set accuracy, while adding little complexity to the typing scheme. However, we saw no benefit to a finer split of hydrogens into four types. Further study, with perhaps a more diverse set of compounds, may be needed to definitively assess whether the finer discrimination among hydrogens by SmirFF.7 and GAFF-1.8, which both have eight hydrogen types, is supported by available experimental data.

Perhaps the second most useful step to improve accuracy is to split oxygen types. When used on its own, it is almost as beneficial as splitting polar from apolar hydrogens, and it may also afford some additional accuracy when used with the hydrogen split. Here, we used the same chemically intuitive oxygen split as in SmirFF.7, but others might be as good or better. Splitting nitrogen types alone did not lead to improved accuracy, though there may be some benefit to splitting nitrogens in the context of the polar/apolar hydrogen split. Interestingly, although GAFF-1.8 lists 13 nitrogen types, they all have identical LJ parameters, and SmirFF.7 has only a single nitrogen type. It should also be mentioned that the improvement due to adding atom types depends on the experimental properties used during training and testing. Also, if other experimental data were used in training, the preferred order of adding atom types might change, and/or other atom splits might become more important.

Using more than one carbon LJ type does not lead to improved accuracy in this study. As in SmirFF.7, our simple LJ typing models considered a split into three-carbon types, based on hybridization state, but this improved results for only training data and it made the test set results worse in most cases, presumably due to overfitting. We did not expect this result, as the current SmirFF.7 and GAFF-1.8 epsilon parameters for carbons have a rather wide range of 0.086 to 0.210 kcal/mol. It may be of interest to revisit these choices and consider dropping back to a single carbon LJ type.

Based on the combined results, an LJ typing model with as few parameters as possible but as many as necessary might be the H2CO3N model, which distinguishes polar and apolar hydrogens and three oxygen types. The accuracy with this model is for all cases similar to or better than even the reoptimized version of SMIRNOFF.

It is also worth mentioning that there are alternative approaches to generating LJ parameters that involve little or no typing. These involve running a quantum mechanical electron structure calculation for the molecule of interest and then computing bespoke LJ parameters for each atom in the molecule as a function of the computed electron density^37,38,44^. These methods may include empirical parameters, which allow the mapping to be tuned so that the LJ parameters yield accurate agreement with reference data, such as the properties of organic liquids considered here.

This study highlights the benefits of direct chemical perception. As implemented in the SMIRNOFF force field specification^28^, this makes introducing, splitting, or combining LJ types as easy as copying a line and modifying a SMIRKS string. It also avoids the complication of other force field specifications where a new LJ type can be added only by adding a new atom type, which in turn mandates adding redundant bonded parameters for the new type. This works both ways, in the sense that adding a new torsion type, for example, also requires adding a new atom type, which in turn needs to be assigned LJ parameters. If one does not actually want to distinguish between the LJ properties of the two atom types, this leads to multiple atom types with identical LJ parameters.

## Methods

### Implementation of Lennard Jones Models

We used the SMIRNOFF FF specification, which uses SMIRKS^45–47^ patterns, to define LJ types, as detailed in Table 3 for all 11 LJ models. The parameter definitions are given as an ordered list of parameter typing rules in ascending order of priority, where each rule consists of a SMIRKS pattern attached to numerical parameters. The top-level (lowest priority) SMIRKS pattern is general (matching any atom of a given element). This parameter is assigned first to all atoms of this element and then overwritten by the more specific SMIRKS patterns, if present. This makes sure that parameters are assigned to all atoms.

**Table 3:** SMIRKS patterns used for LJ typing, with well-depth ε (kcal/mol) and effective radius r_1/2_ (Å) parameter values used to initiate parameter optimization. The first row for each element (bold) gives the parameters used when that element had only a single type. In this case, the SMIRKS pattern was replaced with a SMIRKS string recognizing all atoms of this element. Starting parameters for the polar/apolar hydrogen models are given in the bottom rows. The last column gives the SMIRNOFF LJ type definitions from which these initial values were drawn.

SMIRKS	Description	Initial *ε*	Initial *r*_1/2_	SMIRNOFF SOURCE
Hydrogen				
[#1:1]-[#6X4]	Hydrogen bound to a sp3 Carbon	0.0157	1.4870	[#1:1]-[#6X4]
[#1:1]-[#6X3]	Hydrogen bound to a sp2 Carbon	0.0150	1.4590	[#1:1]-[#6X3]
[#1:1]-[#7]	Hydrogen bound to an Oxygen	0.0157	0.6000	[#1:1]-[#7]
[#1:1]-[#8]	Hydrogen bound to a Nitrogen	5.27E-05	0.3000	[#1:1]-[#8]
Carbon				
[#6X3:1]	sp2 Carbon	0.0860	1.9080	[#6:1]
[#6X4:1]	sp3 Carbon	0.1094	1.9080	[#6X4:1]
[#6X2:1]	sp Carbon	0.2100	1.9080	[#6X2:1]
Oxygen				
[#8X1:1]	Carbonyl Oxygen	0.2100	1.6612	[#8:1]
[#8X2H0 + 0:1]	Alcohol Oxygen	0.1700	1.6837	[#8X2H0 + 0:1]
[#8X2H1 + 0:1]	Ether Oxygen	0.2104	1.7210	[#8X2H1 + 0:1]
Nitrogen				
[#7X1:1]	Nitro Nitrogen	0.1700	1.8240	[#7:1]
[#7X3:1]	Amine Nitrogen	0.1700	1.8240	[#7:1]
Polar and Apolar Hydrogens				
[#1:1] -[#6]	Apolar Hydrogen	0.0157	1.4870	[#1:1]-[#6X4]
[#1:1]-[#7,#8]	Polar Hydrogen	0.0157	0.6000	[#1:1]-[#7]

The parameter definitions are based on the following form of the LJ interaction energy between atoms *i* and *j*:

1 $$E_{LJ,ij} = \varepsilon \left[ {\left( {\frac{{r_{\min }^{12}}}{{r^{12}}}} \right) - \left( {\frac{{2r_{\min }^6}}{{r^6}}} \right)} \right]$$

2 $$\varepsilon = \left( {\varepsilon _{\frak{i}}\varepsilon _j} \right)^{0.5}$$

3 $$r_{\min } = r_{1/2,i} + r_{1/2,j}$$

Here *r* is the interatomic distance, *ε* is the depth of the energy well, *r*_min_ is the minimum energy interatomic distance, *ε*_*i*_, *ε*_*j*_, and *r*_1/2,*i*_, *r*_1/2,*j*_ are the atomic LJ parameters we wish to adjust, and the second and third equations define the Lorentz–Berthelot combining rules.

### Optimization and evaluation of Lennard–Jones parameters

The LJ model optimization procedure is the same as in our previous study^27^ and is summarized in this section. We trained all LJ models against experimentally measured densities and heats of vaporizations of pure organic liquids, properties that have been extensively used in force field parameterization^36,48–50^. The trained models then were tested for their ability to replicate heats of vaporization, densities, and dielectric constants of a separate set of pure organic liquids. The results were compared with matched evaluations of SmirFF.7^28^.

**Fig. 8:**
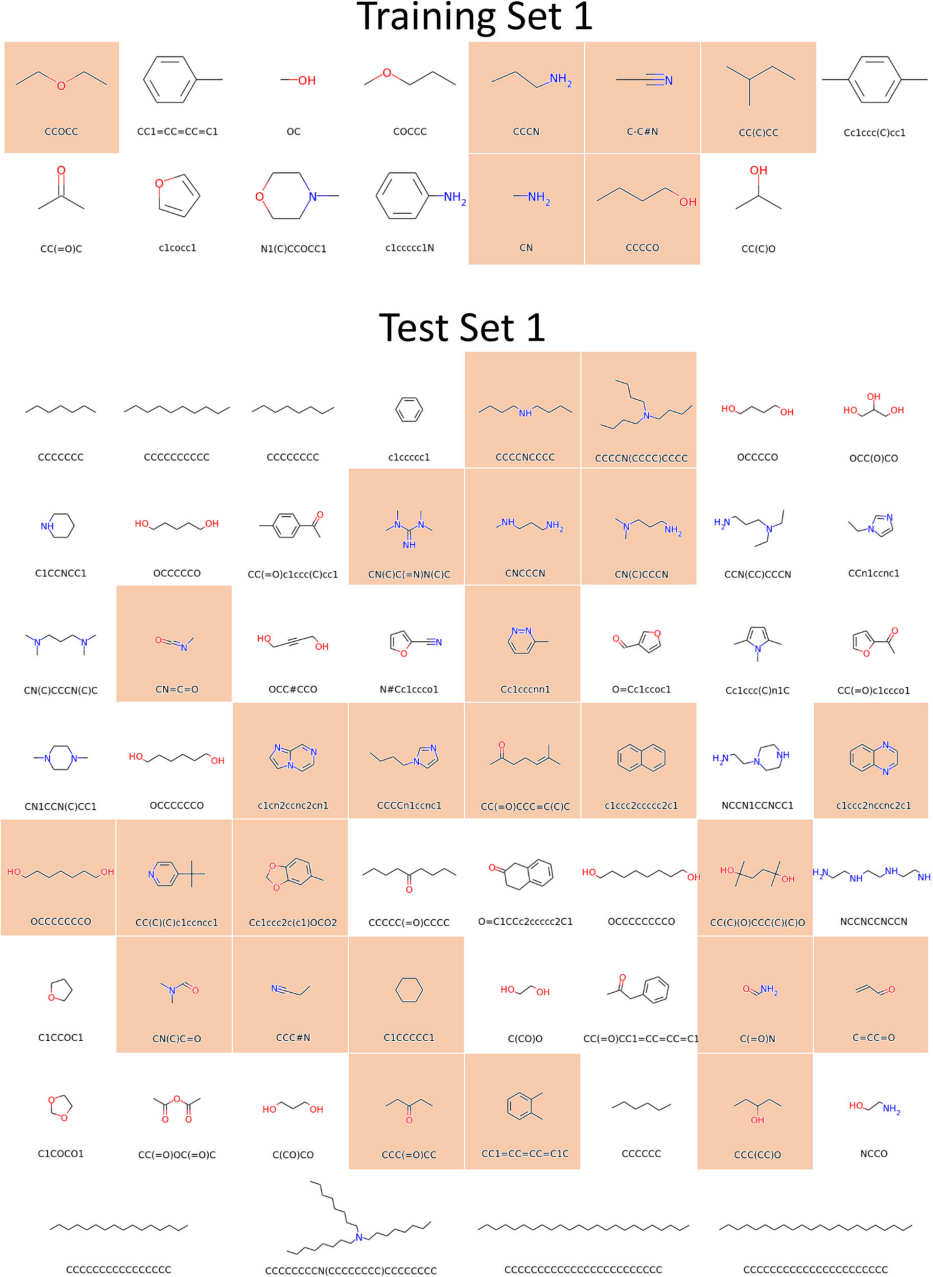
Training and test set compounds used in this study. Training and Test Sets 1 are indicated by the headers. Training Set 2 molecules are designated by the orange squares, and Test Set 2 comprises all molecules on a white background. A SMILES string for each molecule is given under the chemical structure, and the SMILES strings are provided as a text file (Supplementary Data 3) in the Supporting Information.

We optimized and tested each LJ typing model for two different test/train splits of a single collection of 75 compounds (Supplementary Data 3) for which experimental liquid state data are available Fig. 8 and file Supplementary Data 4 in Supporting Information). These compounds span 15 distinct LJ atom types in SmirFF.7 and 28 atom types in GAFF-1.8. Training Set 1 contains 15 molecules (Fig. 8, top) selected to include functional groups, e.g., ether, alcohol, amine, that span all the chemistries in the full set of 75 compounds, and Test Set 1 contains the remaining 60 compounds. Training Set 2 contains 30 compounds drawn at random from the full set of 75 compounds, and Test Set 2 contains the remaining 45. Training set 2 happens to also cover all functional groups. Experimental values for the heats of vaporization of each pure liquid were taken from ThermoML^51^, and densities were taken from ThermoML when available, and otherwise from PubChem^52^. Dielectric constants were taken from multiple sources^53^.

The program ForceBalance^29^ version 1.6.0 was used with the training and test set data described above to optimize the LJ parameters for the LJ typing models in Fig. 1. Each LJ type has two parameters, *r*_1/2_ and *ε*. SmirFF.7 parameters were used as starting values (Table 3)^28^. The ForceBalance objective function used in this study is described in the following. The *N* physical parameters **K** = (*K*_1_, *K*_2_…*K*_*N*_)—here the values of *r*_1/2_ and *ε* for each LJ type—are transformed to mathematical parameters **k** = (*k*_1_, *k*_2_…*k*_*N*_) by shifting and scaling:

4 $$k_i = \frac{1}{{t_i}}\left( {K_i - K_i^o} \right)$$

Here 1/*t*_*i*_ is a scaling factor, also called the prior width, defined as 0.1 kcal/mol for *ε* and 1.0 Å for *r*_1/2_. $K_i^0$ is the initial value of the force field parameter *K*_*i*_. The objective function *L*(**k**) contains a contribution *L*_*m*_(**k**) from each training-set molecule *m*_for_ a training set with *M* molecules. *L*_*m*_(**k**) quantifies the deviation of its *P* computed properties from experiment; Tikhonov regularization with a weighting factor *w*_*reg*_ = 0.1 ensures no extremely large deviations from the starting values:

5 $$L({\mathbf{k}}) = \mathop {\sum}\limits_{m = 1}^M {L_m({\mathbf{k}}) + w_{reg}\left| {\mathbf{k}} \right|^2}$$

6 $$L_m({\mathbf{k}}) = \mathop {\sum}\limits_{p = 1}^P {\frac{1}{{d_p^2}}\left| {y_p^m({\mathbf{k}}) - y_{p,ref}^m} \right|^2}$$

The scaling factors $d_p^m$ balance the weighting of the properties and remove their units; we used *d*_*density*_ = 95 kg/m^3^ and *d*_*HOV*_ = 0.95 kJ/mol for all molecules *m*. Here $y_p^m\left( {\mathbf{k}} \right)$ is the value of the *p*^*th*^ property for molecule *m* (e.g., its density) computed for mathematical parameters **k**, and $y_{p,ref}^m$ is the experimental reference value of this property.

The values of the scaling factors were chosen to afford suitable contributions from both properties to the objective function. Interestingly, although they place greater weight on errors in HOV than on errors in density, the percent errors following fitting are nonetheless greater for HOV than for density (Figs. 2–7).

One could, alternatively, weight the contribution to the objective function of the error in each calculated property as the inverse of the corresponding experimental uncertainty. The logic of this is that it would place more weight on experimental properties that are more certain. However, it is not clear this approach yields the most useful possible force field. For example, if density data were orders of magnitude more reliable than energetic data, such as HOVs, then weighting errors according to experimental uncertainty might yield a force field that was accurate for densities but not for energetic quantities. This expected tradeoff stems at least in part from the fact that the functional form of the force field is crude, so no set of parameters will allow it to accurately replicate all experimental data. If the functional form were more comprehensive, then fitting to one set of experimental observables might improve the accuracy for other observables, but that is not likely to hold when the functional form is fundamentally incapable of replicating all experimental observables with high accuracy.

The optimization was terminated when the step-size for the mathematical parameters fell below 0.01 or the unitless objective function changed <1.0 between two iterations; further details are provided below in this section. All optimizations were performed three times with different random number seeds for the molecular dynamics simulations. The Results section reports the lowest values of the objective function across all three training-set runs and across all three test-sets. The ranges of these quantities across the triplicates are reported in the Supporting Information (Supplementary Data 1).

### Simulation details

ForceBalance calls OpenMM^54^ to compute physical properties from molecular simulations for each iteration of the parameter optimization. For each iteration and each molecule, a gas phase and a liquid phase simulation at T = 298 K were run to calculate liquid state properties and the heat of vaporization. ForceBalance was also used to set up simulations with baseline SmirFF.7 and GAFF1.8 parameters, as well as with optimized parameters for the test set molecules. The gas/liquid difference between the mean potential energy per molecule plus the pressure-volume term RT was used to calculate the heats of vaporization. The mean volumes of the liquid state NPT simulations were used to calculate the liquid state densities. The fluctuations of the simulations box’s dipole moments were used to calculate the dielectric constant.

For all OpenFF-based simulations, the bonded FF terms were drawn from SmirFF.7, and covalent bonds to hydrogen atoms were constrained to their equilibrium lengths with SETTLE (water) and CCMA^55,56^. Calculations with the GAFF-1.8 force field were run for comparison^32,57^. Liquid-phase calculations (700 copies of the molecule of interest) were run for 1.2 ns (0.2 ns equilibration, 1 ns production), with a Langevin integrator. A timestep of 1 fs and a collision frequency of 1 ps^−1^ were applied. A Monte Carlo barostat with a move attempt interval of 25 timesteps was used to maintain the pressure at 1 atm^58^. Long-ranged electrostatics were included via Particle Mesh Ewald summation with a cutoff of 8.5 Å on the short-ranged component. A long-range dispersion correction was applied. The single-molecule gas phase simulations were run for 25 ns (5 ns equilibration, 20 ns production) with a timestep of 1 fs using a Langevin integrator with a collision frequency of 1 ps^−1^ and, infinite distance cutoffs and without periodic boundary conditions. The standard errors of the computed densities, heats of vaporization, and dielectric constants are roughly 0.001 g cc^−1^, 0.3 kJ mol^−1^, and 10%, respectively.

## Supplementary information

Supplementary Information

Descriptions of Additional Supplementary Files

Supplementary Data 1

Supplementary Data 2

Supplementary Data 3

Supplementary Data 4

## Data Availability

The data supporting the findings of this study are available within the article and its Supplementary Information files. Other relevant source data are available at DOI 10.5281/zenodo.3940634 (10.5281/zenodo.3940634), or from the corresponding authors upon reasonable request.

## References

[1] Dror RO (2012). Biomolecular simulation: a computational microscope for molecular biology. Annu. Rev. Biophys..

[2] Shaw DE (2010). Atomic-level characterization of the structural dynamics of proteins. Science.

[3] Abriata LA, Dal M (2015). Peraro, assessing the potential of atomistic molecular dynamics simulations to probe reversible protein-protein recognition and binding. Sci. Rep..

[4] Karplus M, McCammon JA (2002). Molecular dynamics simulations of biomolecules. Nat. Struct. Biol..

[5] Šponer J, Cang X, Cheatham TE (2012). Molecular dynamics simulations of G-DNA and perspectives on the simulation of nucleic acid structures. Methods.

[6] Bergonzo C, Hall KB, Cheatham TE (2015). Stem-loop V of varkud satellite RNA exhibits characteristics of the Mg(2+) bound structure in the presence of monovalent Ions. J. Phys. Chem. B.

[7] Robertson JC, Cheatham TE (2015). DNA backbone Bi/Bii distribution and dynamics in E2 protein-bound environment determined by molecular dynamics simulations. J. Phys. Chem. B.

[8] Wang L, Berne BJ, Friesner RA (2012). On achieving high accuracy and reliability in the calculation of relative protein–ligand binding affinities. Proc. Natl Acad. Sci. USA.

[9] Limongelli V (2012). Funnel metadynamics as accurate binding free-energy method. Proc. Natl Acad. Sci. USA.

[10] Henriksen NM, Fenley AT, Gilson MK (2015). Computational calorimetry: high-precision calculation of host–guest binding thermodynamics. J. Chem. Theory Comput..

[11] Shirts MR, Chodera JD (2008). Statistically optimal analysis of samples from multiple equilibrium states. J. Chem. Phys..

[12] Sugita Y, Okamoto Y (1999). Replica-exchange molecular dynamics method for protein folding. Chem. Phys. Lett..

[13] Muddana HS (2014). The Sampl4 host–guest blind prediction challenge: an overview. J. Computer-aided Mol. Des..

[14] Muddana HS (2014). The sampl4 hydration challenge: evaluation of partial charge sets with explicit-water molecular dynamics simulations. J. Computer-aided Mol. Des..

[15] Nerenberg PS, Head-Gordon T (2018). New developments in force fields for biomolecular simulations. Curr. Opin. Struct. Biol..

[16] Lamoureux G, MacKerell AD, Roux BT (2003). A simple polarizable model of water based on classical drude oscillators. J. Chem. Phys..

[17] Patel S, Brooks CL (2004). Charmm fluctuating charge force field for proteins: I parameterization and application to bulk organic liquid simulations. J. Comput. Chem..

[18] Ponder JW (2010). Current status of the amoeba polarizable force field. J. Phys. Chem. B.

[19] Wang L-P, Chen J, Van T (2013). Systematic parametrization of polarizable force fields from quantum chemistry data. J. Chem. Theory Comput..

[20] Slochower, D., et al., Binding Thermodynamics of Host-Guest Systems with Smirnoff99frosst 1.0.5 from the Open Force Field Initiative. 2019. 10.1021/acs.jctc.9b00748 PMC7328435 31603667

[21] Henriksen NM, Gilson MK (2017). Evaluating force field performance in thermodynamic calculations of cyclodextrin host–guest binding: water models, partial charges, and host force field parameters. J. Chem. Theory Comput..

[22] Yin J (2015). Toward improved force-field accuracy through sensitivity analysis of host-guest binding thermodynamics. J. Phys. Chem. B.

[23] Bell DR (2016). Calculating binding free energies of host–guest systems using the amoeba polarizable force field. Phys. Chem. Chem. Phys..

[24] Skillman AG (2012). Sampl3: blinded prediction of host–guest binding affinities, hydration free energies, and trypsin inhibitors. J. Computer-aided Mol. Des..

[25] Rizzi A (2018). Overview of the sampl6 host–guest binding affinity prediction challenge. J. Computer-aided Mol. Des..

[26] Zhou A, Schauperl M, Nerenberg PS (2020). Benchmarking electronic structure methods for accurate fixed-charge electrostatic models. J. Chem. Inf. Modeling.

[27] Schauperl M (2020). Non-bonded force field model with advanced restrained electrostatic potential charges (Resp2). Commun. Chem..

[28] Mobley D (2018). Escaping atom types in force fields using direct chemical perception. J. Chem. Theory Comput..

[29] Wang L-P, Martinez TJ, Pande VS (2014). Building force fields: an automatic, systematic, and reproducible approach. J. Phys. Chem. Lett..

[30] Wu JC, Chattree G, Ren P (2012). Automation of amoeba polarizable force field parameterization for small molecules. Theor. Chem. Acc..

[31] Zanette C (2019). Toward learned chemical perception of force field typing rules. J. Chem. Theory Comput..

[32] Wang J (2004). Development and testing of a general amber force field. J. Comput Chem..

[33] Lindorff-Larsen K (2010). Improved side-chain torsion potentials for the amber Ff99sb protein force field. Proteins.

[34] Khoury GA, Bhatia N, Floudas CA (2014). Hydration free energies calculated using the amber Ff03 charge model for natural and unnatural amino acids and multiple water models. Comput. Chem. Eng..

[35] Jorgensen WL, Tirado-Rives J (1988). The Opls [Optimized Potentials for Liquid Simulations] potential functions for proteins, energy minimizations for crystals of cyclic peptides and crambin. J. Am. Chem. Soc..

[36] Jorgensen W, Maxwell D, Tirado-Rives J (1996). Development and testing of the Opls all-atom force field on conformational energetics and properties of organic liquids. J. Am. Chem. Soc..

[37] Cole DJ (2016). Biomolecular force field parameterization via atoms-in-molecule electron density partitioning. J. Chem. Theory Comput..

[38] Kantonen SM (2020). Data-driven mapping of gas-phase quantum calculations to general force field Lennard-Jones parameters. J. Chem. Theory Comput..

[39] Bayly CI (1993). A well-behaved electrostatic potential based method using charge restraints for deriving atomic charges: the resp model. J. Phys. Chem..

[40] Dauber-Osguthorpe P, Hagler AT (2019). Biomolecular force fields: where have we been, where are we now, where do we need to go and how do we get there?. J. Computer-aided Mol. Des..

[41] O’Toole, G. Everything Should Be Made as Simple as Possible, but Not Simpler. 2011.

[42] Davis, L., Handbook of Genetic Algorithms. (1991).

[43] Vanderbilt D, Louie SG (1984). A Monte Carlo simulated annealing approach to optimization over continuous variables. J. Computational Phys..

[44] Tkatchenko A, Scheffler M (2009). Accurate molecular Van Der Waals interactions from ground-state electron density and free-atom reference data. Phys. Rev. Lett..

[45] Sayle, R. 1st-Class Smarts Patterns. In EuroMUG 97. (1997).

[46] *Smarts Theory Manual*. Santa Fe, New Mexico.

[47] Weininger D (1988). Smiles, a chemical language and information system. 1. Introduction to methodology and encoding rules. J. Chem. Inf. Computer Sci..

[48] Ryckaert J-P, Bellemans A (1978). Molecular dynamics of liquid alkanes. Faraday Discuss. Chem. Soc..

[49] Jorgensen WL (1981). Quantum and statistical mechanical studies of liquids. 10. Transferable intermolecular potential functions for water, alcohols, and ethers. application to liquid water. J. Am. Chem. Soc..

[50] Vanommeslaeghe K, MacKerell AD (2015). Charmm additive and polarizable force fields for biophysics and computer-aided drug design. Biochim. Biophys. Acta (BBA) - Gen. Subj..

[51] Frenkel M (2006). Xml-based iupac standard for experimental, predicted, and critically evaluated thermodynamic property data storage and capture (Thermoml)(Iupac Recommendations 2006). Pure Appl. Chem..

[52] Kim S (2018). Pubchem 2019 update: improved access to chemical data. Nucleic Acids Res..

[53] Lide, D. R. *CRC Handbook of Chemistry and Physics: A Ready-Reference Book of Chemical and Physical Data*. (CRC-Press, 1995).

[54] Eastman P (2013). Openmm 4: a reusable, extensible, hardware independent library for high performance molecular simulation. J. Chem. Theory Comput..

[55] Eastman P, Pande VS (2010). Ccma: a robust, parallelizable constraint method for molecular simulations. J. Chem. Theory Comput..

[56] Miyamoto S, Kollman PA (1992). Settle: an analytical version of the shake and rattle algorithm for rigid water models. J. Comput. Chem..

[57] Mackerell AD (2004). Empirical force fields for biological macromolecules: overview and issues. J. Comput. Chem..

[58] Chow K-H, Ferguson DM (1995). Isothermal-Isobaric molecular dynamics simulations with Monte Carlo volume sampling. Computer Phys. Commun..

