# Supplementary material for: Data-driven analysis of the number of Lennard–Jones types needed in a force field
Source: Commun Chem. 2020 Nov 13;3:173. doi: 10.1038/s42004-020-00395-w (PMC8294475; doi:10.1038/s42004-020-00395-w)
Supplement: Supplementary file 1 — Supplementary Information [file 42004_2020_395_MOESM1_ESM.pdf]

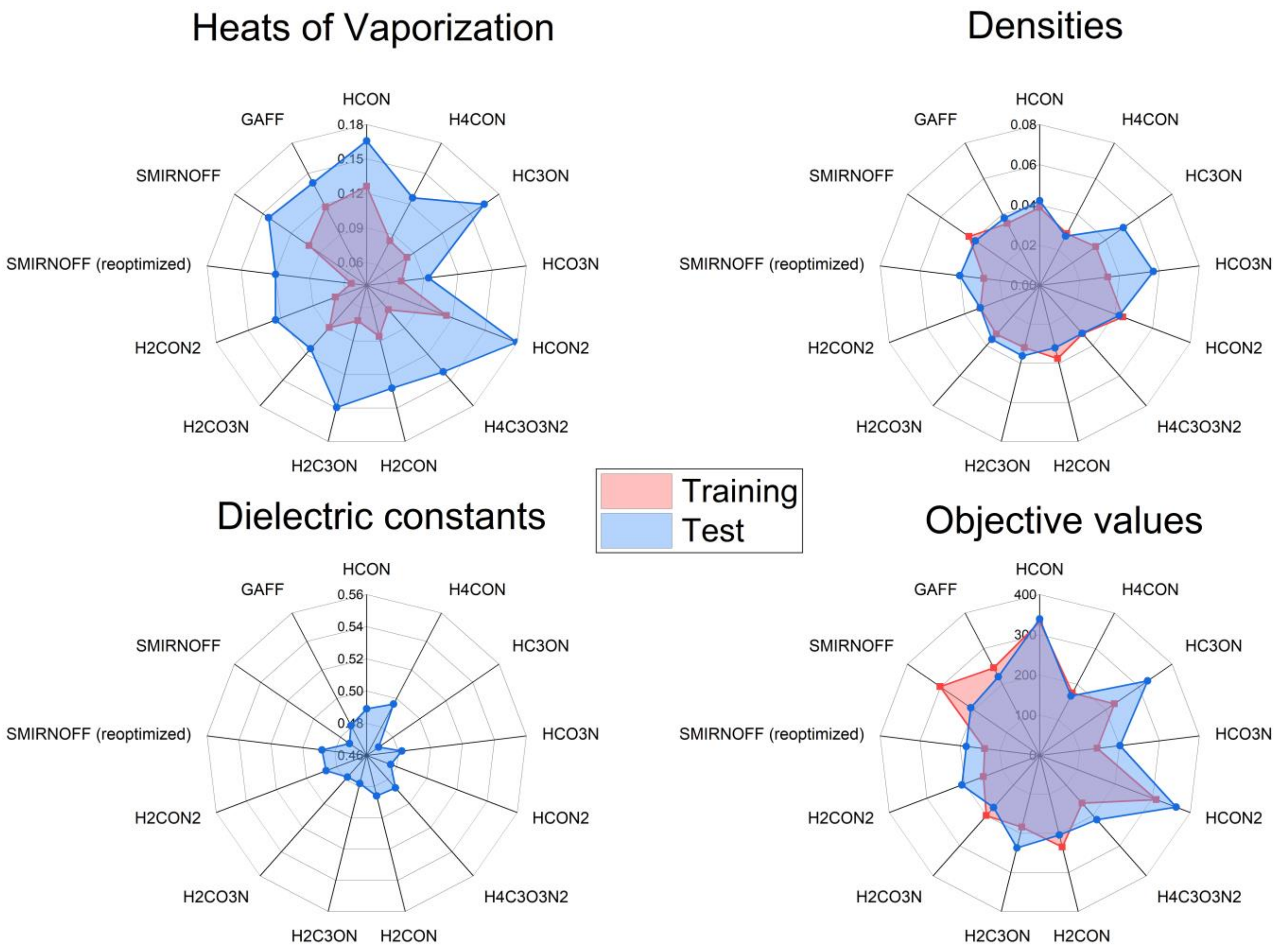

Supplementary Figure 1: Relative errors for Training and Test Set 1 with RESP2 charges. *Comparison of relative errors for heats of vaporization, density, and dielectric constant, and ForceBalance objective function, for Training Set 1 (red) and Test Set 1 (blue) sets in the context of the RESP2 charge model. The test set objective functions are scaled by a factor of 1/15.*

LJ types	LJ model	Mean Error Density %	Mean Error Heats of Vaporization %	Objective Function
H C O N	HCON	3.87%	12.62%	332
H H H H C O N	H4CON	2.91%	8.38%	175
H C C C O N	HC3ON	3.39%	8.27%	226
H C O O O N	HCO3N	3.41%	7.05%	144
H C O N N	HCON2	4.43%	11.43%	310
H H H H C C C O O O N N	H4C3O3N2	3.22%	6.85%	159
H H C O N	H2CON	3.75%	8.59%	235
H H C C C O N	H2C3ON	3.19%	7.18%	184
H H C O O O N	H2CO3N	3.25%	8.90%	200
H H C O N N	H2CON2	3.16%	6.91%	150
H H H H H H H H C C C O O O N	SMIRNOFF (reoptimized)	2.80%	5.34%	138
H H H H H H H H C C C O O O N	SMIRNOFF	4.27%	10.08%	301
H H H H H H H H H H H C C C C C O O O N N	GAFF	3.48%	11.69%	246

Supplementary Figure 2: Training set results for Training Set 1 optimization of LJ parameters with RESP2 partial charges. *Errors and objective function values are reported for the Training Set 1 compounds. These results are for the replicates that gave minimum training set values of the objective function for each model.*

LJ types	LJ model	Mean Error Density %	Mean Error Heats of Vaporization %	Dielectric constants %	Objective Function
H C O N	HCON	4.21%	16.57%	48.9%	339
H H H H C O N	H4CON	2.77%	12.60%	49.6%	167
H C C C O N	HC3ON	5.05%	16.44%	46.9%	326
H C O O O N	HCO3N	5.69%	9.43%	48.2%	201
H C O N N	HCON2	4.23%	17.95%	47.6%	363
H H H H C C C O O O N N	H4C3O3N2	3.19%	14.05%	48.7%	214
H H C O N	H2CON	3.20%	13.22%	48.6%	204
H H C C C O N	H2C3ON	3.62%	14.94%	47.8%	237
H H C O O O N	H2CO3N	3.59%	11.37%	47.8%	173
H H C O N N	H2CON2	3.16%	12.47%	48.7%	207
H H H H H H H H C C C O O O N	SMIRNOFF (reoptimized)	4.00%	11.98%	48.8%	184
H H H H H H H H C C C O O O N	SMIRNOFF	3.89%	14.38%	47.3%	208
[illegible]	GAFF	3.78%	14.08%	48.1%	221

Supplementary Figure 3: Test set results for Training Set 1 optimization of LJ parameters with RESP2 partial charges*. Errors and objective function values are reported for the Test Set 1 compounds. Objective function values are scaled by 1/15 (see main text). These results are for the replicates that gave minimum training set values of the objective function for each model.*

Supplementary Table 1: Force field vectors for optimized LJ models. "Scaled epsilon": epsilon values scaled up by the ratio of the mean of $r_{1/2}$ to the mean of epsilon, 13.69, to put epsilon and $r_{1/2}$ on a similar footing.

	**Unscaled epsilon**								**Scaled epsilon**					
**SMIRKS**		**HCON**	**HCO3N**	**H2CON**	**H2CO3N**	**H2CO3Nreopt**				**HCON**	**HCO3N**	**H2CON**	**H2CO3N**	**H2CO3Nreopt**
[#1:1]-[#6X4]	$r_{1/2}$	1.27	1.42	1.39	1.42	1.42			$r_{1/2}$	1.27	1.42	1.39	1.42	1.42
[#1:1]-[#6X3]	$r_{1/2}$	1.27	1.42	1.39	1.42	1.42			$r_{1/2}$	1.27	1.42	1.39	1.42	1.42
[#1:1]-[#7]	$r_{1/2}$	1.27	1.42	0.69	0.88	1.44			$r_{1/2}$	1.27	1.42	0.69	0.88	1.44
[#1:1]-[#8]	$r_{1/2}$	1.27	1.42	0.69	0.88	1.44			$r_{1/2}$	1.27	1.42	0.69	0.88	1.44
[#6:1]	$r_{1/2}$	2.11	1.84	2.06	1.96	1.87			$r_{1/2}$	2.11	1.84	2.06	1.96	1.87
[#6X4:1]	$r_{1/2}$	2.11	1.84	2.06	1.96	1.87			$r_{1/2}$	2.11	1.84	2.06	1.96	1.87
[#6X2:1]	$r_{1/2}$	2.11	1.84	2.06	1.96	1.87			$r_{1/2}$	2.11	1.84	2.06	1.96	1.87
[#7X3:1]	$r_{1/2}$	1.47	1.79	1.8	1.83	1.78			$r_{1/2}$	1.47	1.79	1.8	1.83	1.78
[#7:1]	$r_{1/2}$	1.47	1.79	1.8	1.83	1.78			$r_{1/2}$	1.47	1.79	1.8	1.83	1.78
[#8:1]	$r_{1/2}$	1.11	1.73	1.61	1.71	1.65			$r_{1/2}$	1.11	1.73	1.61	1.71	1.65
[#8X2H1+0:1]	$r_{1/2}$	1.11	1.89	1.61	1.6	1.87	**Mean**		$r_{1/2}$	1.11	1.89	1.61	1.6	1.87
[#8X2H0+0:1]	$r_{1/2}$	1.11	1.82	1.61	1.59	1.82	1.599		$r_{1/2}$	1.11	1.82	1.61	1.59	1.82
[#1:1]-[#6X4]	epsilon	0.017	0.034	0.014	0.023	0.03			epsilon*13.68	0.23	0.47	0.19	0.31	0.41
[#1:1]-[#6X3]	epsilon	0.017	0.034	0.014	0.023	0.03			epsilon*13.68	0.23	0.47	0.19	0.31	0.41
[#1:1]-[#7]	epsilon	0.017	0.034	0.015	0.007	0.031			epsilon*13.68	0.23	0.47	0.21	0.10	0.42
[#1:1]-[#8]	epsilon	0.017	0.034	0.015	0.007	0.031			epsilon*13.68	0.23	0.47	0.21	0.10	0.42
[#6:1]	epsilon	0.074	0.077	0.081	0.078	0.078			epsilon*13.68	1.01	1.05	1.11	1.07	1.07
[#6X4:1]	epsilon	0.074	0.077	0.081	0.078	0.078			epsilon*13.68	1.01	1.05	1.11	1.07	1.07
[#6X2:1]	epsilon	0.074	0.077	0.081	0.078	0.078			epsilon*13.68	1.01	1.05	1.11	1.07	1.07
[#7X3:1]	epsilon	0.194	0.231	0.177	0.21	0.245			epsilon*13.68	2.66	3.16	2.42	2.87	3.35
[#7:1]	epsilon	0.194	0.231	0.177	0.21	0.245			epsilon*13.68	2.66	3.16	2.42	2.87	3.35
[#8:1]	epsilon	0.22	0.137	0.205	0.197	0.1			epsilon*13.68	3.01	1.88	2.81	2.70	1.37
[#8X2H1+0:1]	epsilon	0.22	0.494	0.205	0.218	0.533	**Mean**		epsilon*13.68	3.01	6.76	2.81	2.98	7.29
[#8X2H0+0:1]	epsilon	0.22	0.087	0.205	0.151	0.096	0.116833		epsilon*13.68	3.01	1.19	2.81	2.07	1.31
						**ratio of means**	13.68616							
