# Supplementary material for: Data-driven analysis of the number of Lennard–Jones types needed in a force field
Source: Commun Chem. 2020 Nov 13;3:173. doi: 10.1038/s42004-020-00395-w (PMC8294475; doi:10.1038/s42004-020-00395-w)
Supplement: Supplementary file 2 — Descriptions of Additional Supplementary Files [file 42004_2020_395_MOESM2_ESM.pdf]

Descriptions of Additional Supplementary Files

Supplementary Data 1

Description: Means and ranges over the triplicate optimizations.

Supplementary Data 2

Description: Optimized LJ parameter sets.

Supplementary Data 3

Description: SMILES strings of compounds in training and test sets.

Supplementary Data 4

Description: Experimental data for training and test set compounds.
